# Regenerative High Purity Type I Collagen Scaffold for Breast Cancer Excision Repair and Reconstruction: A Prospective Multicenter Clinical Trial

**DOI:** 10.7759/cureus.105608

**Published:** 2026-03-21

**Authors:** Ravi Krishnappa, Naveen Narayan, Ashwini Narasimhamurthy, Sumith S Deep

**Affiliations:** 1 Department of Surgical Oncology, JSS Academy of Higher Education and Research, Mysuru, IND; 2 Department of Plastic Reconstructive and Aesthetic Surgery, Adichunchanagiri Institute of Medical Sciences, B. G. Nagara, IND; 3 Department of Anaesthesiology, JSS Academy of Higher Education and Research, Mysuru, IND; 4 Department of Surgical Oncology, JSS Hospital, Mysuru, IND

**Keywords:** breast-conserving surgery, breast-q outcomes, breast volume restoration, collagen scaffold, high-purity type i collagen, mri integration, oncoplastic breast reconstruction, partial breast defect reconstruction, regenerative biomaterial, tissue regeneration

## Abstract

Background: Breast-conserving surgery (BCS) is the preferred treatment for early-stage breast cancer; however, excision-related volume loss frequently results in contour deformity, asymmetry, and reduced patient satisfaction. Conventional volume displacement and replacement techniques are limited by donor-site morbidity, technical complexity, and unpredictable aesthetic outcomes. High-purity type I collagen (HPTC) scaffolds represent a regenerative, biocompatible strategy designed to provide immediate volume support while promoting host tissue integration.

Methods: This prospective, single-arm, multicenter clinical trial was conducted at two tertiary care centers in India. Forty women undergoing BCS with an anticipated postoperative volume loss ≥20% received intraoperative implantation of an HPTC scaffold into the excision cavity. Patients were followed for two months. The primary outcome was breast volume restoration success, defined as ≥80% objective volume retention at two months with patient satisfaction ≥7/10. Secondary outcomes included objective volumetric analysis using MRI and three-dimensional surface imaging, radiologic integration assessed by the MRI Integration Score (MIS), cosmetic outcomes measured using the BREAST-Q, and safety. Longitudinal outcomes were analyzed using repeated-measures statistics, with effect sizes calculated to assess clinical relevance.

Results: All 40 enrolled patients completed the two-month follow-up. The composite primary endpoint (≥80% volume retention plus patient satisfaction ≥7/10) was achieved in 82.5% of patients (33/40). Mean breast volume retention at two months was 88.6% ± 9.8%, significantly exceeding the predefined benchmark of 75% (p < 0.001; Cohen's d = 1.39). MIS demonstrated progressive scaffold integration, improving from 3.2 ± 1.1 postoperatively to 9.1 ± 1.4 at eight weeks (p < 0.001; Cohen's d = 2.8), with no suspicious imaging features. BREAST-Q "Satisfaction with Breasts" scores improved by 24.7 points from baseline (54.2 ± 8.6 to 78.9 ± 7.4; p < 0.001; Cohen's d = 1.8). No device-related serious adverse events occurred; minor complications (seroma 10%, infection 5%) resolved with conservative management.

Conclusion: HPTC scaffold implantation following BCS demonstrates excellent safety, robust early volume restoration (88.6% retention at two months), progressive biological integration, and clinically meaningful improvement in patient satisfaction. This minimally invasive regenerative approach avoids donor-site morbidity while achieving outcomes comparable to traditional oncoplastic techniques, warranting evaluation in randomized controlled trials with longer follow-up to assess durability and radiotherapy response.

## Introduction

Breast-conserving surgery (BCS) has become the standard surgical approach for early-stage breast cancer, offering oncologic outcomes equivalent to mastectomy while preserving the native breast mound. Despite its advantages, excision of tumor-bearing tissue frequently results in postoperative volume deficits, contour deformities, and asymmetry, which can significantly compromise cosmetic outcomes and patient quality of life. These deformities are particularly impactful in small- to moderate-volume breasts, where even modest tissue loss may produce visible distortion, compared to the contralateral side [1].

Traditional strategies for managing post-BCS volume loss include volume displacement techniques, regional or local flaps, autologous fat grafting, and, less commonly, implant-based approaches. While effective in selected cases, these methods are associated with important limitations. Flap-based reconstruction increases operative complexity and donor-site morbidity, fat grafting is limited by unpredictable resorption and the need for repeat procedures, and implants are generally unsuitable for partial defects and may interfere with radiotherapy and oncologic surveillance [2]. Consequently, there remains an unmet clinical need for minimally invasive, biologically compatible solutions capable of restoring volume while preserving breast architecture and oncologic safety.

The ideal volume restoration strategy following breast cancer resection in BCS would combine several key features: (1) immediate structural support to prevent cavity collapse, (2) biological integration promoting neovascularization and tissue regeneration, (3) minimal additional surgical morbidity, (4) compatibility with adjuvant radiotherapy, (5) preservation of imaging interpretability for cancer surveillance, and (6) predictable, durable cosmetic outcomes. Currently, no single technique fulfills all these criteria, highlighting a critical gap in oncoplastic breast surgery that regenerative biomaterials may address.

Regenerative medicine approaches have gained increasing attention as alternatives to traditional reconstruction. Among these, collagen-based scaffolds have emerged as promising tools due to their ability to provide immediate structural support while simultaneously promoting host tissue integration, neovascularization, and gradual remodeling. Type I collagen, the principal structural protein of the extracellular matrix, plays a central role in wound healing and tissue regeneration by supporting fibroblast migration, angiogenesis, and organized tissue deposition [3,4].

An evolution in collagen biomaterials, un-crosslinked high-purity type I collagen (HPTC) with added bioactivity through phosphorylation, is characterized by reduced antigenicity, predictable biodegradation, and reliable tissue integration. Extensive clinical experience with HPTC-based constructs in complex wound environments has demonstrated their capacity to facilitate durable tissue regeneration across a range of indications. Early work describing novel collagen application techniques over meshed split-thickness skin grafts highlighted improved graft stability and wound coverage in challenging defects, establishing the biological versatility of collagen scaffolds in surgical reconstruction [1]. Subsequent clinical series demonstrated the successful use of collagen-based matrices as alternatives to flap coverage for exposed bone and tendon, further supporting their load-bearing and regenerative capabilities [2].

Randomized controlled trials conducted by our group and others have consistently shown superior or comparable outcomes with HPTC-based skin substitutes compared with dehydrated human amnion/chorion membrane (dHACM) in diabetic foot ulcers, venous leg ulcers, and pressure ulcers, with advantages in healing rate, tissue quality, and safety profile [3-7]. These findings have been reinforced by multicenter trials and systematic reviews demonstrating favorable histopathological remodeling, angiogenesis, and epithelialization with HPTC use in chronic wounds [8]. Beyond wound care, collagen-based biomaterials have also shown efficacy in complex surgical scenarios, including the management of infected prosthetic meshes, where collagen matrices facilitated tissue incorporation and infection resolution without foreign-body complications [9].

Collectively, this body of evidence underscores the biological consistency of HPTC scaffolds across diverse tissue environments, suggesting potential applicability beyond surface wounds to deeper soft-tissue defects. The breast excision cavity following BCS represents a uniquely suitable target for such regenerative scaffolds, as it requires immediate volume support, predictable integration, compatibility with adjuvant radiotherapy, and preservation of imaging interpretability for cancer surveillance [10,11].

Despite encouraging preclinical and early clinical data on collagen-based fillers for soft-tissue reconstruction, prospective human studies evaluating HPTC scaffolds specifically for breast volume restoration after BCS remain limited [12,13]. Furthermore, standardized radiologic and volumetric outcome measures assessing scaffold integration and volume maintenance in the breast are lacking. Addressing these gaps is essential to establishing the clinical role of regenerative scaffolds within oncoplastic breast surgery.

The present prospective, multicenter clinical trial was therefore designed to evaluate the safety, efficacy, radiologic integration, volumetric stability, cosmetic outcomes, and patient-reported satisfaction associated with implantation of an HPTC scaffold for immediate volume restoration following BCS. By integrating objective imaging-based metrics with validated patient-reported outcomes, this study aims to provide comprehensive evidence supporting the use of HPTC scaffolds as a regenerative alternative to conventional volume-replacement techniques in breast cancer surgery.

## Materials and methods

Study design and setting

This prospective, single-arm, multicenter clinical trial was conducted at two tertiary care academic institutions in Karnataka, India: JSS Medical College and Hospital, Mysuru, and Adichunchanagiri Institute of Medical Sciences (AIMS), B. G. Nagara. The study was designed to evaluate the safety, volumetric efficacy, radiologic integration, cosmetic outcomes, and patient-reported satisfaction following implantation of an HPTC scaffold for immediate volume restoration after breast cancer resection surgery. The trial was registered prospectively with ClinicalTrials.gov (ID: NCT07360730) and approved by the Institutional Ethics Committee, AIMS, B. G. Nagara (Approval No.: AIMS/IEC/240/2025, dated October 15, 2025).

The trial was conducted in accordance with the principles of the Declaration of Helsinki and Good Clinical Practice guidelines. Approval was obtained from the Institutional Ethics Committees of both participating centers prior to patient enrollment. Written informed consent was obtained from all participants. The methodological framework, outcome hierarchy, and statistical rigor were modeled on previously published multicenter HPTC trials to ensure internal validity and reproducibility.

Patient selection and eligibility criteria

Women aged 18 to 70 years undergoing BCS for stage 0-II breast carcinoma or high-risk premalignant breast lesions were screened for eligibility. Patients were required to have a unifocal lesion suitable for wide local excision with an anticipated postoperative breast volume deficit of at least 20%, as estimated intraoperatively by the operating surgeon. Adequate skin envelope viability and the ability to undergo serial MRI examinations were mandatory.

Exclusion criteria included multicentric disease requiring mastectomy, prior radiotherapy to the ipsilateral breast, active local or systemic infection, known hypersensitivity to collagen-based products, autoimmune or connective tissue disorders, uncontrolled systemic illness, pregnancy or lactation, and inability to comply with postoperative follow-up or patient-reported outcome assessments.

Sample size determination

This study was designed as a prospective feasibility and early efficacy trial. Sample size was determined based on precision estimation for the primary composite endpoint rather than traditional hypothesis testing. The required sample size (n) for estimation of a proportion with specified precision was determined using the standard formula:

$n = \frac{Z^2 \, p (1 - p)}{d^2}$

where p is the anticipated success proportion (0.75), d is the desired precision (0.125), and Z = 1.96 for a 95% confidence interval. This yielded a minimum sample of 36; allowing for attrition, 40 patients were enrolled.

Assuming a true success rate of 75% (based on oncoplastic literature), a sample of 40 patients provides an expected 95% confidence interval width of approximately 25%, which was deemed acceptable for preliminary efficacy assessment and effect size estimation to inform future definitive trials. The sample size also ensures adequate power (>80%) to detect a clinically meaningful difference of 15% in volume retention compared to the benchmark of 75%, assuming a standard deviation of 10% and α = 0.05.

Surgical technique and intervention

All patients underwent standard oncologic BCS performed by experienced breast or plastic surgeons at the respective centers. Following tumor excision with confirmed hemostasis and margin adequacy, the excision cavity was carefully assessed for volume deficit and three-dimensional geometry.

**Figure 1:**
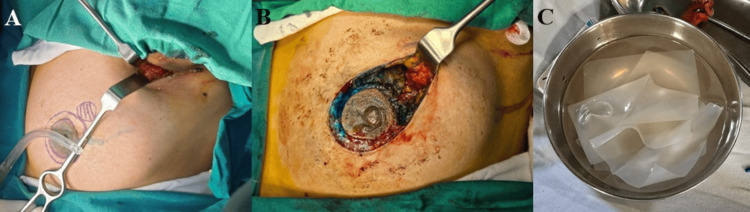
Intraoperative steps of high-purity type I collagen scaffold–assisted breast volume restoration following breast cancer excision (A) Skin marking of the breast tumour with concurrent axillary dissection in progress; (B) Post-excision defect in the upper outer quadrant demonstrating the residual cavity after tumour resection; (C) Hydration and preparation of the high-purity type I collagen scaffold prior to implantation

**Figure 2:**
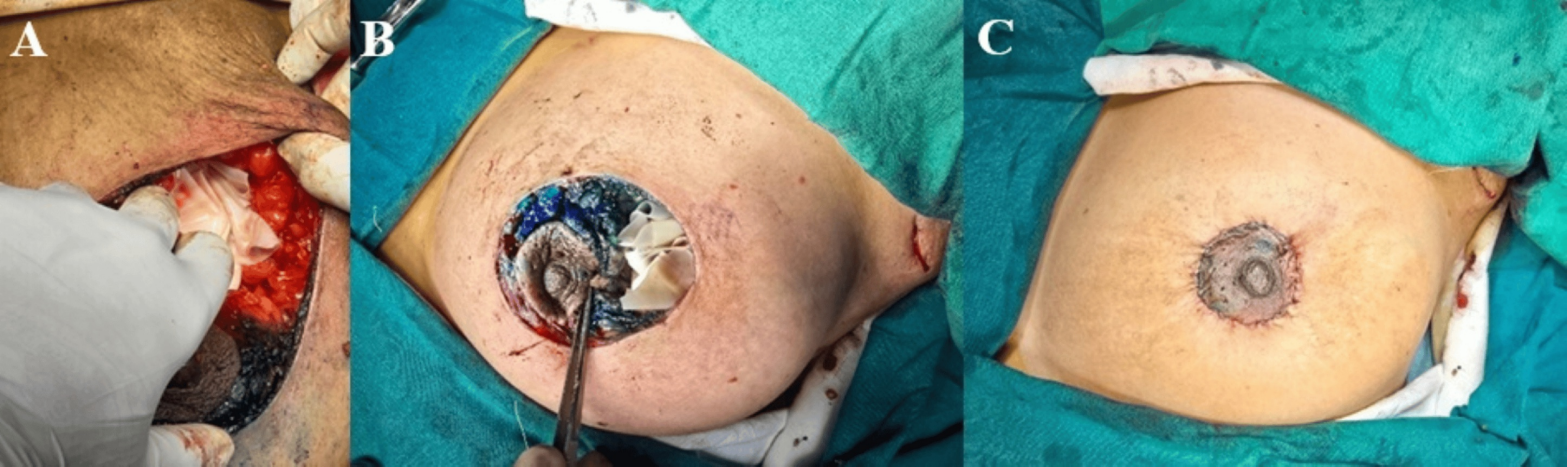
Intraoperative steps of high-purity type I collagen scaffold–assisted breast volume restoration following breast cancer resection (A) Placement of the high-purity type I collagen scaffold into the excision cavity to restore volume; (B) Scaffold fixation secured with absorbable sutures to surrounding breast parenchyma; (C) Completed layered skin closure following scaffold implantation

An HPTC scaffold was trimmed intraoperatively to conform precisely to the dimensions of the surgical cavity and implanted without tension. The scaffold was positioned in direct contact with surrounding viable breast tissue to facilitate cellular infiltration and neovascularization. No additional fixation beyond absorbable sutures was routinely required. Layered parenchymal and skin closure was performed using standard techniques. The use of surgical drains was left to the discretion of the operating surgeon based on oncologic considerations rather than scaffold placement (Figures 1, 2). Postoperative management, including antibiotics, wound care, chemotherapy, and radiotherapy, followed institutional breast cancer treatment protocols.

Primary outcome: breast volume restoration success

The primary efficacy endpoint was breast volume restoration success at two months postoperatively, defined as a composite outcome incorporating both objective volumetric preservation and patient-perceived cosmetic satisfaction. Success required achievement of at least 80% objective breast volume retention relative to the immediate postoperative baseline, together with a patient satisfaction score of ≥7 on a 10-point Likert scale. This composite endpoint was selected to ensure that structural volume preservation translated into clinically meaningful cosmetic benefit and patient acceptance.

Objective breast volume retention

Objective breast volume retention was assessed using a dual-modality approach comprising three-dimensional surface photogrammetry and magnetic resonance imaging (MRI) volumetry. Baseline measurements were obtained within 48 hours postoperatively and repeated at two, four, and eight weeks. Breast volumes were calculated for the operated breast and normalized against the immediate postoperative volume to derive percentage volume retention. MRI volumetry served as the reference standard, while 3D surface imaging provided adjunctive validation and assessment of symmetry.

Volume retention was expressed as a continuous variable and analyzed longitudinally to assess temporal stability and controlled scaffold resorption.

MRI Integration Score (MIS)

Radiologic integration of the HPTC scaffold was evaluated using a predefined MIS, a composite semi-quantitative scoring system developed for this study based on established MRI criteria for biomaterial integration and soft-tissue regeneration reported in prior scaffold and regenerative filler studies [7,9]. MRI examinations were performed immediately postoperatively and at four and eight weeks using standardized T1-weighted, T2-weighted, and dynamic contrast-enhanced sequences.

The MIS evaluated four domains: scaffold-host tissue integration, neovascularization based on enhancement patterns, scaffold resorption and volume replacement, and tissue quality within the scaffold region. Each domain was scored from 0 to 3, yielding a total score ranging from 0 to 12, with higher scores indicating superior integration and regenerative remodeling.

Cosmetic outcome: BREAST-Q

Cosmetic outcomes were assessed using the BREAST-Q questionnaire [14], a validated patient-reported outcome measure specific to breast surgery. Permission to use the BREAST-Q questionnaire was obtained from the original developer. The “Satisfaction with Breasts” domain was prespecified as the primary cosmetic endpoint. Questionnaires were administered preoperatively and at four and eight weeks postoperatively. Raw scores were converted to standardized scores ranging from 0 to 100 according to BREAST-Q scoring manuals, with higher scores indicating greater satisfaction [14].

Improvement over baseline and attainment of an absolute score ≥70 at final follow-up were considered clinically meaningful cosmetic outcomes.

Safety and complications

Safety assessment included prospective recording of all adverse events and postoperative complications, including seroma, surgical site infection, hematoma, wound dehiscence, scaffold migration or exposure, and delays in adjuvant oncologic therapy. Device-related serious adverse events were specifically monitored. All events were managed according to institutional protocols and documented throughout the follow-up period.

Data collection and follow-up

Baseline demographic data, tumor characteristics, operative details, and immediate postoperative imaging were recorded at enrollment. Follow-up assessments were conducted at two, four, and eight weeks postoperatively, during which volumetric measurements, MRI evaluation, BREAST-Q questionnaires [14], and complication surveillance were completed. Data were recorded in standardized case record forms at both centers to ensure uniformity.

Statistical analysis plan

Statistical analysis was performed using IBM SPSS Statistics, version 28.0 (IBM Corp., Armonk, NY, USA). Continuous variables were summarized as mean ± standard deviation, while categorical variables were expressed as frequencies and percentages. Longitudinal changes in breast volume retention, MIS, and BREAST-Q scores were analyzed using repeated-measures analysis of variance. One-sample t-tests were used to compare observed volume retention against predefined clinical benchmarks. Correlations between objective volumetric outcomes and patient-reported satisfaction were assessed using Pearson correlation coefficients. A two-sided p value of less than 0.05 was considered statistically significant.

## Results

Study population and baseline characteristics

**Figure 3:**
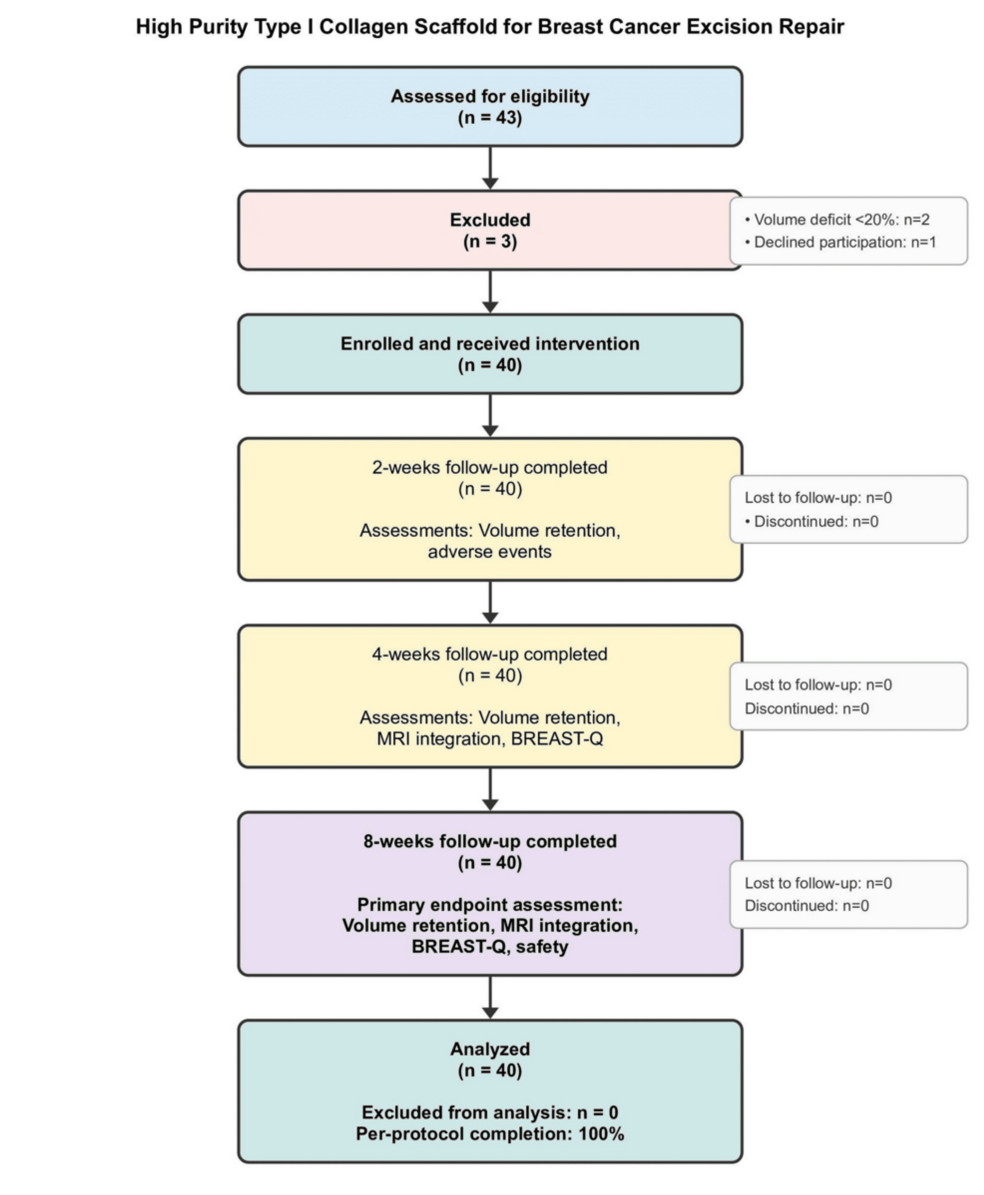
CONSORT (Consolidated Standards of Reporting Trials) diagram Of 43 patients assessed, 40 were enrolled and received the intervention; three were excluded (volume deficit <20%, n=2; declined participation, n=1). All treated patients completed two, four, and eight-week follow-up. Primary endpoint (≥80% volume retention with BREAST-Q satisfaction ≥7) was achieved in 82.5% (33/40). Participating centers: Adichunchanagiri Institute of Medical Sciences, B. G. Nagara, Karnataka, India; JSS Medical College and Hospital, Mysuru, India.

A total of 43 patients were screened for eligibility across the two participating centers. Three patients were excluded: two due to anticipated volume deficit <20% and one who declined participation. Forty patients were enrolled and underwent HPTC scaffold implantation. All 40 patients (100%) completed the planned two-month follow-up without protocol deviations or loss to follow-up (Figure 3).

Baseline demographic and tumor characteristics are summarized in Table 1. The mean age of participants was 49.6 ± 8.9 years (range: 32-68 years), and the mean body mass index was 24.8 ± 3.6 kg/m². All patients had an Eastern Cooperative Oncology Group (ECOG) performance status of 0 or 1 at enrollment.

**Table 1:** Baseline demographic and clinical characteristics (n=40) Baseline demographic and tumor characteristics of the study population ECOG: Eastern Cooperative Oncology Group; DCIS: ductal carcinoma in situ

Characteristic		Value
Age, years (mean ± SD)		49.6 ± 8.9
Age range, years		32-68
Body mass index, kg/m² (mean ± SD)		24.8 ± 3.6
ECOG performance status, n (%)	0	28 (70.0)
	1	12 (30.0)
Histopathological diagnosis, n (%)	Invasive ductal carcinoma	28 (70.0)
	Ductal carcinoma in situ	8 (20.0)
	High-risk benign / premalignant	4 (10.0)
Tumor stage, n (%)	Stage 0 (DCIS)	8 (20.0)
	Stage I	22 (55.0)
	Stage II	10 (25.0)
Tumor location, n (%)	Upper outer quadrant	25 (62.5)
	Upper inner quadrant	8 (20.0)
	Lower outer quadrant	5 (12.5)
	Lower inner quadrant	2 (5.0)
Excised specimen volume, mL (mean ± SD)		112 ± 34
Estimated volume deficit, % (mean ± SD)		24.6 ± 5.2
Planned adjuvant therapy, n (%)	Chemotherapy	15 (37.5)
	Radiotherapy	38 (95.0)
	Endocrine therapy	30 (75.0)

Histopathological diagnosis revealed invasive ductal carcinoma in 70% (28/40) of patients, ductal carcinoma in situ in 20%, and high-risk benign or premalignant lesions in 10%. Tumor staging demonstrated early disease predominance, with 75% of patients classified as stage 0 or I. The mean excised specimen volume was 112 ± 34 mL, corresponding to a mean estimated postoperative volume deficit of 24.6% of total breast volume. The upper outer quadrant was the most commonly involved region (62.5%).

Primary outcome: breast volume restoration success

At two months postoperatively, 33 of 40 patients (82.5%) met the predefined composite primary endpoint of breast volume restoration success, defined as objective breast volume retention ≥80% together with patient satisfaction ≥7/10. This high success rate reflects concordance between structural volume preservation and patient-perceived cosmetic benefit.

The mean objective volume retention at eight weeks was 88.6% ± 9.8, significantly exceeding the predefined clinical benchmark of 75% (mean difference: 13.6%; 95% CI: 9.8-17.4; p < 0.001). Mean patient satisfaction on the 10-point Likert scale was 8.1 ± 1.2, further supporting the composite success outcome.

The primary outcome analysis demonstrated a high rate of successful breast volume restoration at two months, with a composite success rate of 82.5% (33 out of 40 patients). When compared with the predefined clinical benchmark success expectation of 60%, derived from published outcomes of fat grafting and conventional oncoplastic techniques, this observed success rate represents a clinically and statistically meaningful improvement. Effect size analysis using Cohen’s h for proportions yielded a value of 0.53, which corresponds to a large effect size.

Objective breast volume retention

Objective volumetric analysis demonstrated robust early volume preservation with gradual, controlled reduction over time, consistent with scaffold remodeling rather than volume collapse. Mean volume retention was 92.4% ± 7.1 at two weeks, 90.1% ± 8.6 at four weeks, and 88.6% ± 9.8 at eight weeks (Table 2).

**Table 2:** Objective breast volume retention over follow-up Objective breast volume retention percentages measured at serial postoperative time points

Time point	Volume retention (%) mean ± SD	95% CI	p-value vs. 75%
2 weeks	92.4 ± 7.1	90.1–94.7	<0.001
4 weeks	90.1 ± 8.6	87.2–93.0	<0.001
8 weeks	88.6 ± 9.8	85.4–91.8	<0.001

Repeated-measures analysis of variance confirmed a statistically significant change in volume retention across follow-up time points (p = 0.032); however, the magnitude of decline was clinically modest, and all mean values remained well above the success threshold. One-sample t-tests demonstrated that volume retention at all time points was significantly greater than the historical benchmark of 75% (p < 0.001 for all comparisons).

**Figure 4:**
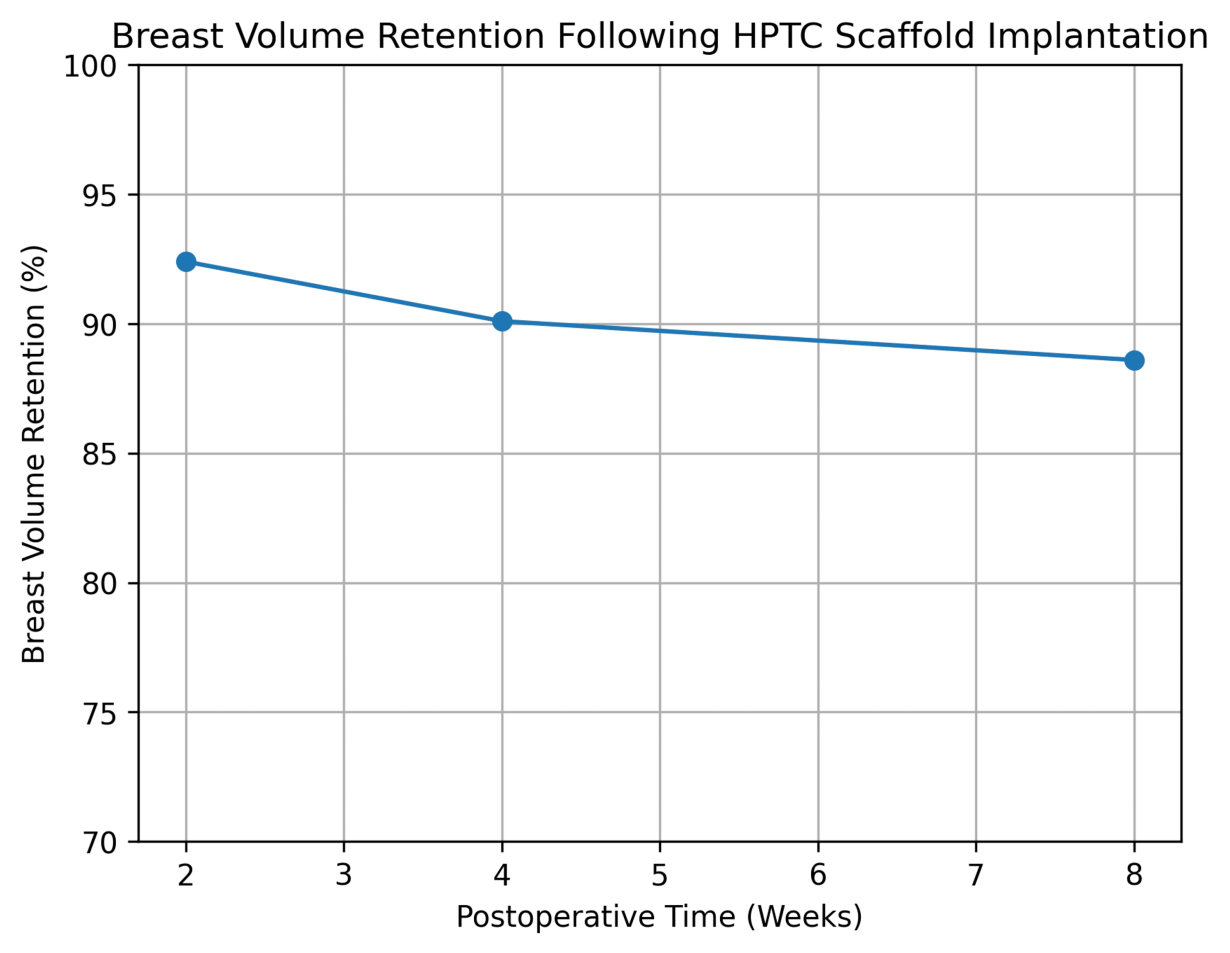
Objective breast volume retention over time Mean percentage breast volume retention at two, four, and eight weeks after scaffold implantation, demonstrating high early preservation with gradual, controlled remodeling HPTC: high-purity type I collagen

These findings are illustrated in Figure 4, which depicts a shallow slope of volume reduction, suggesting early volumetric stabilization.

At eight weeks postoperatively, the mean objective breast volume retention was 88.6% ± 9.8, notably exceeding the established historical benchmark of 75%. This corresponds to a mean difference of 13.6%, indicating superior volumetric preservation with the intervention. One-sample effect size analysis demonstrated a very large effect (Cohen’s d = 1.39), confirming that the observed improvement was not only statistically significant but also of substantial clinical importance.

Radiologic integration: MRI Integration Score (MIS)

MRI analysis demonstrated progressive biological integration of the HPTC scaffold over time. The mean MIS increased from 3.2 ± 1.1 immediately postoperatively to 6.8 ± 1.3 at four weeks and 9.1 ± 1.4 at eight weeks, indicating a transition from early structural placement to advanced tissue integration and neovascularization (Table 3).

**Table 3:** MRI (Magnetic Resonance Imaging) Integration Score (MIS) progression MIS progression demonstrating scaffold integration and remodeling

Time point	MIS (mean ± SD)	p-value vs. baseline
Immediate postoperative	3.2 ± 1.1	–
4 weeks	6.8 ± 1.3	<0.001
8 weeks	9.1 ± 1.4	<0.001

**Figure 5:**
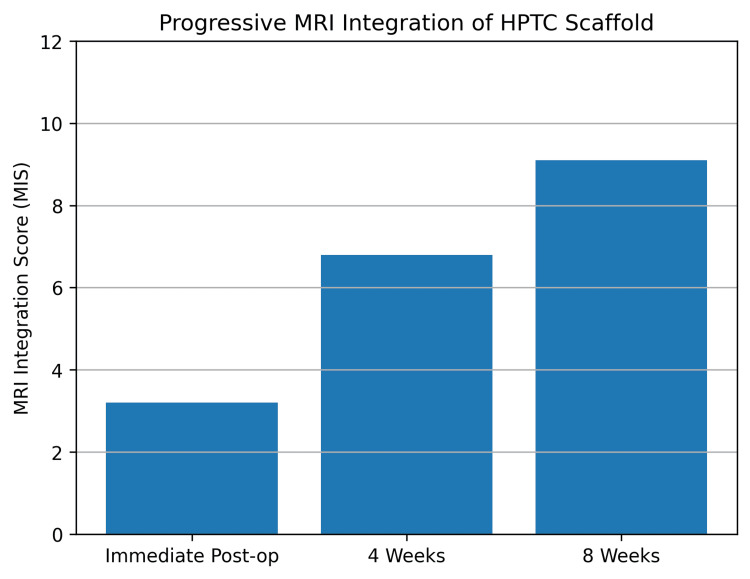
MRI (Magnetic Resonance Imaging) Integration Score (MIS) progression Longitudinal improvement in MIS from immediate postoperative imaging to eight weeks, indicating progressive scaffold–host tissue integration and neovascularization HPTC: high-purity type I collagen

Repeated-measures analysis confirmed a highly significant improvement in MIS across time points (p < 0.001). Dynamic contrast-enhanced MRI revealed increasing early-phase enhancement within the scaffold region, consistent with neovascularization, without suspicious mass-like enhancement or features suggestive of local recurrence. The trajectory of MIS improvement is depicted in Figure 5.

The MIS demonstrated a marked and sustained improvement over the postoperative follow-up period. Mean MIS values increased from 3.2 ± 1.1 immediately after surgery to 9.1 ± 1.4 at eight weeks, corresponding to a mean increase of 5.9 points. This change was associated with an extremely large paired effect size (Cohen’s d = 2.8), indicating a highly robust and consistent treatment effect across participants.

Cosmetic outcome: BREAST-Q

Patient-reported cosmetic satisfaction demonstrated significant and clinically meaningful improvement over the study period. The mean BREAST-Q “Satisfaction with Breasts” score [14] increased from a preoperative baseline of 54.2 ± 8.6 to 72.5 ± 7.9 at four weeks and 78.9 ± 7.4 at eight weeks (Table 4).

**Table 4:** BREAST-Q "Satisfaction with Breasts" scores BREAST-Q "Satisfaction with Breasts" scores across follow-up visits

Time point	Mean score ± SD
Preoperative	54.2 ± 8.6
4 weeks	72.5 ± 7.9
8 weeks	78.9 ± 7.4

**Figure 6:**
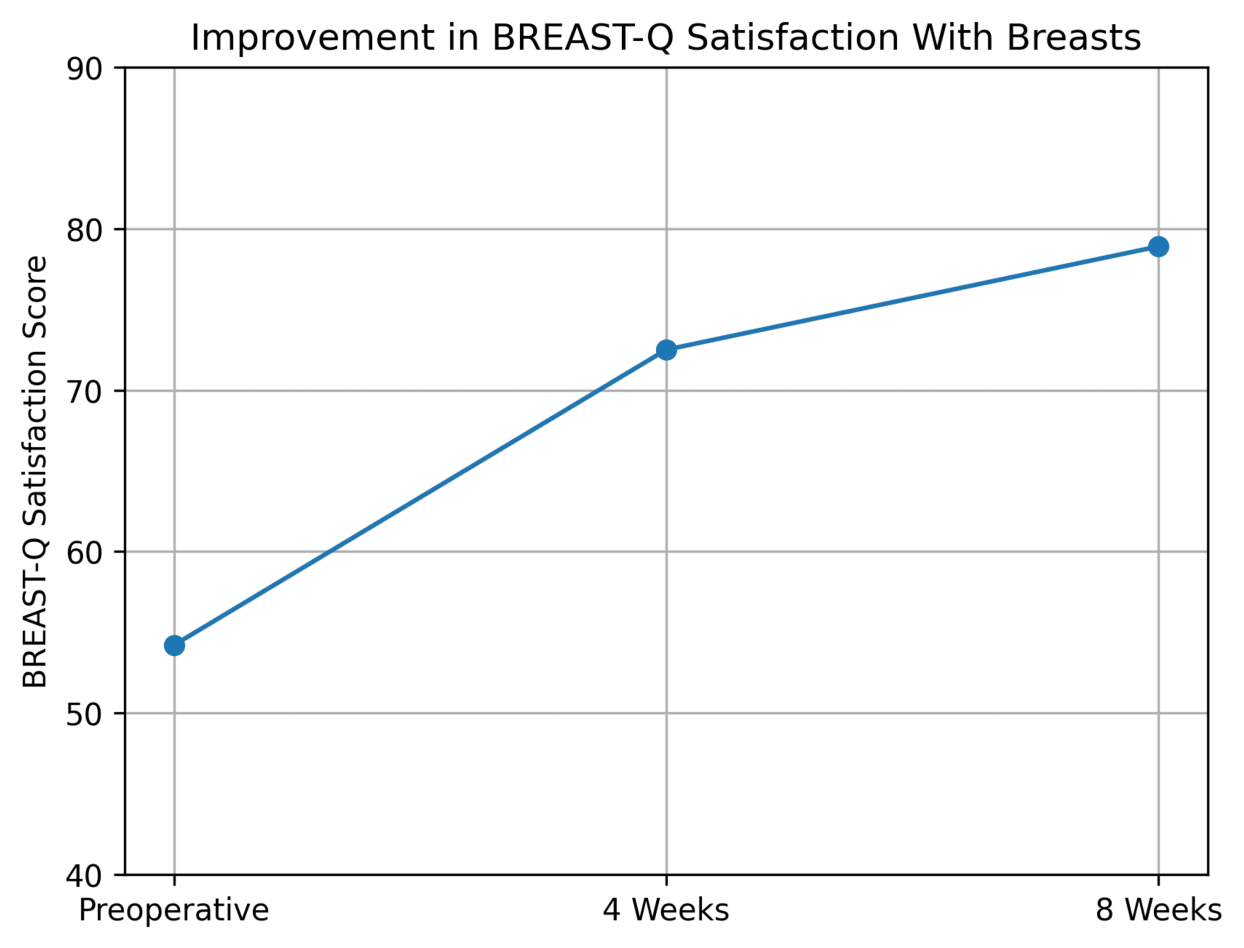
BREAST-Q "Satisfaction with Breasts" scores Patient-reported cosmetic satisfaction (BREAST-Q domain) measured preoperatively and during follow-up, demonstrating clinically meaningful improvement after scaffold-assisted reconstruction.

The mean improvement from baseline to eight weeks was +24.7 points, representing a large effect size and exceeding thresholds for clinically meaningful change. Repeated-measures analysis confirmed statistically significant improvement across all time points (p < 0.001). At final follow-up, 87.5% of patients achieved an absolute BREAST-Q score ≥70. These improvements are graphically represented in Figure 6.

Physician-rated cosmetic assessment at two months classified outcomes as excellent in 55% of patients, good in 35%, fair in 10%, and poor in none, supporting concordance between patient-reported and clinician-assessed outcomes.

Patient-reported cosmetic outcomes demonstrated a substantial improvement over the study period. BREAST-Q “Satisfaction with Breasts” scores increased by 24.7 points from baseline to eight weeks postoperatively. This change corresponded to a large effect size (Cohen’s d = 1.8), indicating a strong and consistent improvement across participants.

Correlation between objective and patient-reported outcomes

Pearson correlation analysis demonstrated a moderate positive correlation between objective volume retention at eight weeks and BREAST-Q satisfaction scores (r = 0.62, p < 0.001), suggesting that structural volume preservation contributes significantly to patient-perceived cosmetic outcomes. However, the correlation was not perfect, indicating that additional factors beyond volume retention - such as breast shape, symmetry, and scar quality - also influence patient satisfaction. MIS at eight weeks showed weak correlation with BREAST-Q scores (r = 0.31, p = 0.052), suggesting that radiologic integration, while biologically important, may not be directly perceived by patients.

Safety and complications

No device-related serious adverse events were observed during the study period. Minor complications occurred in a limited number of patients and were managed conservatively. Seroma formation was noted in four patients (10%), superficial surgical site infection in two patients (5%), and transient postoperative pain requiring prolonged analgesia in six patients (15%). No cases of hematoma, scaffold migration, scaffold exposure, wound dehiscence, or delay in adjuvant oncologic therapy were recorded. Table 5 provides comprehensive safety data demonstrating the excellent tolerability profile of HPTC scaffold implantation. All observed complications were minor (Common Terminology Criteria for Adverse Events (CTCAE) grade 1-2) and resolved completely with conservative management [15].

**Table 5:** Detailed adverse events and management * Postoperative complications and safety outcomes observed during the study period SSI: superficial surgical site infection; NSAIDs: non‑steroidal anti‑inflammatory drugs; SAE: serious adverse event

Adverse event	N (%)	Grade* [15]	Time to onset (days)	Management	Resolution time (days)	Relation to device
Minor complications						
Seroma	4 (10.0)	1-2	8.5 ± 3.2	Aspiration (n=3) Observation (n=1)	14.2 ± 6.1	Possibly related
Superficial SSI	2 (5.0)	1	5.0 ± 1.4	Oral antibiotics	7.5 ± 2.1	Unrelated
Prolonged pain	6 (15.0)	1	Immediate	Extended NSAIDs	21.3 ± 8.7	Possibly related
Ecchymosis	3 (7.5)	1	Immediate	Observation	12.0 ± 3.5	Unrelated
Major complications						
Hematoma requiring evacuation	0	-	-	-	-	-
Deep SSI/abscess	0	-	-	-	-	-
Scaffold exposure	0	-	-	-	-	-
Wound dehiscence	0	-	-	-	-	-
Device-related SAE	0	-	-	-	-	-
Delay in adjuvant therapy	0	-	-	-	-	-

Table 6 presents exploratory subgroup analyses to identify patient characteristics that may influence treatment success. The composite primary endpoint (≥80% volume retention plus patient satisfaction ≥7/10) was achieved in 82.5% of the overall population, with notable variation across subgroups. The only statistically significant predictor of outcome was volume deficit magnitude (p = 0.038). Patients with smaller defects (20-25% volume loss) achieved 92.0% success compared to 66.7% in those with larger defects (>25%). This finding suggests that HPTC scaffolds perform optimally in moderate volume deficits, and larger defects may require supplementary techniques such as combination with volume displacement procedures or larger scaffold volumes.

**Table 6:** Subgroup analysis of primary endpoint by patient characteristics #Chi-square or Fisher's exact test; *Statistically significant (p < 0.05) DCIS: ductal carcinoma in situ

Subgroup	n	Primary endpoint success n (%)	p-value^#^
Overall	40	33 (82.5%)	-
Age			0.421
<50 years	22	19 (86.4%)	-
≥50 years	18	14 (77.8%)	
BMI			0.156
<25 kg/m²	24	21 (87.5%)	-
≥25 kg/m²	16	12 (75.0%)	
Volume deficit			0.038*
20-25%	25	23 (92.0%)	-
>25%	15	10 (66.7%)	
Tumor location			0.612
Upper outer quadrant	25	21 (84.0%)	-
Other quadrants	15	12 (80.0%)	
Histology			0.287
DCIS	8	8 (100%)	-
Invasive carcinoma	28	22 (78.6%)	
Benign/premalignant	4	3 (75.0%)	

The study was powered as a feasibility and early efficacy trial with emphasis on effect size estimation rather than hypothesis testing alone. Given the single-arm design, comparisons were made against clinically relevant historical benchmarks rather than a concurrent control group. Effect sizes were calculated to quantify the magnitude of observed outcomes and to support clinical interpretability beyond p values. The consistently large effect sizes observed across volumetric, radiologic, and patient-reported outcomes provide strong justification for the biological and clinical relevance of the intervention and support progression to future randomized controlled trials.

## Discussion

Breast-conserving surgery (BCS) has evolved from a procedure focused solely on oncologic outcomes to one in which aesthetic results and patient quality of life are recognized as equally important treatment goals, reflecting the shift toward patient-centered cancer care. Contemporary oncoplastic frameworks emphasize the importance of restoring breast volume and contour while preserving oncologic safety, particularly in small- to moderate-sized breasts where excision-related deformity is most pronounced [1-3]. Despite advances in volume displacement and replacement techniques, an unmet need persists for minimally invasive, reproducible solutions that restore volume without increasing surgical morbidity or compromising postoperative surveillance [14,16,17].

The primary efficacy endpoint was breast volume restoration success through effective healing of cancer excision wound sites by scaffold integration postoperatively, as measured using MIS at two months, which was assessed by both objective volumetric preservation and patient-perceived cosmetic satisfaction, reflecting not only breast volume restoration but also effective healing, following cancer excision. The observed volumetric stability and progressive radiologic integration can be attributed to the unique biological properties of HPTC scaffolds. Type I collagen serves as the primary structural component of the extracellular matrix and provides a biomimetic template that actively promotes cellular infiltration, angiogenesis, and tissue remodeling. The un-crosslinked HPTC scaffold with added bioactivity through phosphorylation achieves a critical balance: sufficient structural integrity to provide immediate volume support and resist premature collapse upon proper healing of the wound, while maintaining porosity and biodegradability that permit cellular ingrowth and gradual replacement with host tissue.

Dynamic contrast-enhanced MRI findings in this study revealed progressive enhancement patterns consistent with neovascularization rather than avascular encapsulation, a hallmark of successful biomaterial integration. This vascular Surgicoll-Mesh® (EnColl Corporation, Fremont, CA, USA; distributed by Advanced Biotech Products Pvt Ltd, Chennai, India) ingrowth is essential for nutrient delivery, waste removal, and sustained tissue viability, ultimately determining the long-term fate of regenerated tissue. The extremely large effect size observed in MIS improvement (Cohen's d = 2.8) reflects not merely passive tolerance of the scaffold but active biological remodeling-a process fundamental to durable reconstruction.

Furthermore, the absence of foreign-body giant cell reactions or fibrous encapsulation, inferred from the lack of rim enhancement or T2-hyperintense capsule formation on MRI, supports the biocompatibility of HPTC scaffolds and their capacity for host tissue integration. These findings align with histopathological studies of HPTC in chronic wound healing, which have documented organized collagen deposition, mature vascular networks, and minimal inflammatory infiltrate at late time points.

The present prospective multicenter study demonstrates that implantation of an HPTC scaffold following BCS results in robust early volume preservation, progressive biological integration, and meaningful improvement in patient-reported cosmetic satisfaction, with a low complication profile and effective cancer excision wound healing. Importantly, these outcomes were achieved without donor-site morbidity, prolonged operative time, or interference with adjuvant oncologic therapy, positioning HPTC scaffolds as a regenerative alternative within the oncoplastic armamentarium.

Clinical significance of volumetric outcomes

Objective volumetric analysis revealed mean breast volume retention of 88.6% at two months, with a very large effect size relative to established historical benchmarks. In contrast, autologous fat grafting (widely used for partial breast defects) is associated with unpredictable resorption rates ranging from 30% to 50% and often necessitates repeat procedures [18]. Volume replacement flaps, while durable, are associated with donor-site morbidity, increased operative complexity, and variable aesthetic outcomes, particularly in smaller breasts [6,10,11].

The controlled and modest decline in volume observed in this study likely reflects physiologic scaffold resorption with concurrent tissue replacement, rather than structural failure. This pattern is particularly advantageous in the breast, where abrupt volume loss translates directly into contour deformity and patient dissatisfaction. The observed volumetric stability supports the role of HPTC scaffolds as a structural-biological hybrid capable of bridging the gap between immediate volume restoration and long-term tissue regeneration.

Radiologic integration and oncologic safety

Progressive improvement in the MIS, with extremely large effect sizes, provides objective radiologic evidence of scaffold-host tissue integration, neovascularization, and remodeling. Dynamic contrast-enhanced MRI demonstrated enhancement patterns consistent with vascular ingrowth rather than encapsulation or foreign-body reaction, findings that parallel regenerative filler behavior described in prior preclinical and translational studies [7].

Equally important, no suspicious enhancement patterns or imaging artifacts compromising oncologic surveillance were observed. This is a critical distinction from fat necrosis and oil cyst formation following lipofilling, which may complicate postoperative imaging and generate patient anxiety. Preservation of imaging interpretability reinforces the oncologic safety of collagen-based scaffolds in breast reconstruction.

Cosmetic and patient-reported outcomes

Patient-reported cosmetic outcomes, assessed using the BREAST-Q [14], demonstrated a large and clinically meaningful improvement over baseline. The magnitude of BREAST-Q score improvement observed in this study compares favorably with reported outcomes following oncoplastic techniques and immediate reconstruction, where patient satisfaction is strongly influenced by contour symmetry, breast feel, and avoidance of visible deformity [14,19].

The concordance between objective volumetric outcomes, radiologic integration, and patient satisfaction underscores the validity of the composite primary endpoint used in this study. This integrated approach aligns with modern standards in breast surgery, which emphasize patient-centered outcomes alongside traditional surgical metrics [3].

Positioning HPTC scaffolds within oncoplastic practice

Volume replacement oncoplastic techniques using perforator flaps or regional tissue remain effective but are technically demanding and not universally applicable [10,11]. Meta-analyses comparing oncoplastic techniques with conventional BCS highlight improved aesthetic outcomes but also increased operative complexity and resource utilization [2]. In contrast, HPTC scaffold implantation represents a simpler, reproducible adjunct that can be integrated into standard BCS workflows without specialized flap expertise.

The biological performance of HPTC scaffolds observed in this study is consistent with extensive clinical experience in complex wound healing, where HPTC has demonstrated superior tissue regeneration, angiogenesis, and safety compared with alternative biological matrices [4,5,12,13]. These consistent regenerative properties across tissue environments support the translational application of HPTC scaffolds to breast reconstruction.

Comparison with existing literature

Oncoplastic BCS has been widely adopted to mitigate the aesthetic sequelae of tumor excision, with techniques broadly categorized into volume displacement and volume replacement approaches [1-3]. Volume displacement techniques are effective for small defects but are limited in small- to moderate-sized breasts, where reshaping alone may be insufficient to correct contour deformity [6]. Volume replacement techniques, including local perforator flaps and regional tissue transfer, offer durable volume restoration but are associated with increased operative complexity, donor-site morbidity, and variability in aesthetic outcomes [10,11].

Meta-analyses comparing standard BCS with oncoplastic approaches demonstrate improved cosmetic outcomes but also highlight heterogeneity in techniques and outcomes, underscoring the need for standardized, reproducible solutions [2,11]. In this context, the present study introduces a regenerative, scaffold-based strategy that differs fundamentally from tissue transfer by leveraging host-mediated tissue regeneration rather than tissue redistribution or replacement.

Autologous fat grafting remains a commonly employed adjunct for partial breast reconstruction; however, evidence-based reviews consistently report unpredictable resorption, fat necrosis, and calcifications that may compromise oncologic imaging and necessitate repeat procedures [18]. In contrast, the HPTC scaffold evaluated in this study demonstrated controlled volume remodeling with consistently high early volume retention and progressive radiologic integration, without imaging artifacts or surveillance concerns.

The volumetric and radiologic findings of the present trial align with regenerative scaffold behavior reported by Puls et al., who demonstrated progressive tissue ingrowth and vascularization using a regenerative tissue filler for BCS in preclinical and early clinical settings [7]. Similarly, Donnelly et al., in their systematic review of tissue engineering approaches to breast reconstruction, emphasized the potential advantages of collagen-based scaffolds in promoting neovascularization and gradual tissue replacement, particularly when compared with inert synthetic implants [9].

Experience with absorbable biosynthetic scaffolds in breast reconstruction, primarily in post-mastectomy settings, has also demonstrated acceptable safety and aesthetic outcomes, supporting the broader concept of scaffold-mediated breast reconstruction [8]. However, unlike post-mastectomy reconstruction, partial breast defects following BCS require precise volume restoration, compatibility with radiotherapy, and preservation of native breast architecture. The present study specifically addresses this clinical niche.

Patient-reported outcomes in this trial, measured using the BREAST-Q, showed large and clinically meaningful improvements that compare favorably with reported outcomes following oncoplastic surgery and immediate reconstruction [14,19]. The concordance between objective volumetric preservation, MRI-based integration, and patient satisfaction strengthens the argument that scaffold-assisted regeneration can achieve outcomes comparable to more invasive techniques, while minimizing morbidity.

Importantly, the biological performance observed in the breast mirrors the consistent regenerative efficacy of HPTC demonstrated in chronic wound management, pressure ulcers, venous leg ulcers, and diabetic foot ulcers [4,5,12,13]. This cross-indication consistency supports the translational robustness of HPTC scaffolds and reinforces their applicability to breast tissue regeneration [20-22].

Comparison with traditional methods of volume restoration

Traditional approaches to volume restoration following BCS can be broadly categorized into autologous fat grafting, volume displacement techniques, and volume replacement using flaps or implants. While each has a defined role, all are associated with inherent limitations.

Autologous fat grafting is widely practiced but is characterized by unpredictable resorption rates, often requiring multiple sessions to achieve durable correction. Fat necrosis, oil cyst formation, and microcalcifications can complicate postoperative imaging and generate diagnostic uncertainty, particularly in oncologic follow-up [18]. Volume displacement techniques rely on reshaping remaining breast tissue and are effective only for small defects or in larger breasts, with limited applicability in many patients [1,6].

Volume replacement using local or regional flaps provides durable tissue but at the cost of donor-site morbidity, increased operative complexity, and prolonged recovery [10,11]. Implant-based approaches are generally unsuitable for partial defects and may interfere with radiotherapy and long-term surveillance.

Published systematic reviews of autologous fat grafting report volume retention typically ranging from approximately 55% to 70%, with revision procedures required in up to 30-50% of cases due to resorption or contour irregularity [18]. In contrast, oncoplastic volume replacement flaps demonstrate high volume durability (approximately 85-95%) but are associated with donor-site morbidity rates of 5-15% and substantially longer operative times [11]. These comparative data from the literature contextualize the present findings, in which HPTC scaffold implantation achieved 88.6% early volume retention without donor-site morbidity or major complications.

In contrast, HPTC scaffold implantation avoids these limitations by providing immediate structural support without tissue transfer, while simultaneously enabling biologic integration and gradual tissue replacement [23-25]. The controlled remodeling observed in this study suggests that scaffold-assisted regeneration may reduce or eliminate the need for secondary corrective procedures, thereby avoiding the cumulative morbidity associated with traditional techniques.

Justification for future randomized controlled trials

While the findings of this study are compelling, the single-arm design and short-term follow-up limit definitive comparative conclusions. However, the consistently large effect sizes across volumetric, radiologic, and patient-reported outcomes provide strong justification for progression to a randomized controlled trial. Future studies should compare HPTC scaffold-assisted reconstruction with established techniques such as fat grafting or volume displacement oncoplastic surgery, with longer follow-up to assess durability, radiation response, and long-term cosmetic stability.

Incorporation of validated quality-of-life instruments such as the EORTC QLQ-C30 (European Organisation for Research and Treatment of Cancer Quality of Life Questionnaire‑Core 30) and EQ-5D (EuroQol 5‑Dimension) would further strengthen comparative effectiveness analyses and align with international standards for oncologic outcomes research [16,17].

Surgicoll-Mesh® - HPTC used in this study

Surgicoll-Mesh® (HPTC scaffold) is an implantable, bioresorbable, type I collagen matrix derived from purified bovine dermal collagen and engineered to function as a regenerative soft-tissue scaffold. The material undergoes proprietary purification and phosphorylation processing to reduce antigenicity while preserving native collagen fibrillar architecture and porosity, thereby facilitating cellular infiltration, neovascularization, and gradual host-tissue remodeling. Surgicoll-Mesh® provides temporary structural support and is progressively replaced by autologous connective tissue during healing [26].

Strengths and limitations of the study

A key strength of this study is its prospective, multicenter design, which enhances generalizability and reduces single-institution bias. Conducting the trial across two tertiary care centers with standardized protocols strengthens external validity and reflects real-world surgical practice. The use of objective, multimodal outcome measures, including MRI volumetry, three-dimensional surface imaging, and a predefined MIS, provides robust and reproducible assessment of scaffold performance beyond subjective aesthetic evaluation. Another major strength is the incorporation of validated patient-reported outcome measures, particularly the BREAST-Q, which aligns the study with contemporary standards emphasizing patient-centered outcomes in breast surgery [14]. The use of a composite primary endpoint integrating objective volume retention with patient satisfaction further ensures that structural success translates into meaningful clinical benefit. The study also benefits from explicit reporting of effect sizes, allowing assessment of clinical relevance beyond statistical significance and facilitating comparison with existing literature. The absence of device-related serious adverse events and lack of interference with oncologic surveillance or adjuvant therapy further reinforces the safety profile of the intervention.

However, several limitations warrant consideration. First, the single-arm design without a concurrent control group limits causal inference and prevents direct statistical comparison with alternative reconstruction techniques. While outcomes were benchmarked against historical controls from the published literature, selection bias, publication bias, and differences in patient populations, surgical techniques, and outcome assessment methods may confound such comparisons. A randomized controlled trial with contemporaneous comparators is essential to establish comparative effectiveness definitively. Second, the two-month follow-up duration, while adequate for assessing early safety and volume retention, is insufficient to evaluate long-term outcomes critical to clinical practice. Key unanswered questions include: (1) volume stability beyond the initial remodeling phase, (2) scaffold behavior and cosmetic outcomes following adjuvant radiotherapy, (3) durability of patient satisfaction over years rather than weeks, (4) long-term oncologic surveillance feasibility, and (5) rates of late complications such as chronic seroma or calcification. An extended follow-up of this cohort, ideally for a minimum of two to five years, is planned and will be reported separately. Third, the study population was relatively homogeneous, consisting of women with early-stage disease (stage 0-II), moderate volume deficits (mean 24.6%), and predominantly upper outer quadrant tumors. Applicability to larger defects (>30% volume loss), central or inferior quadrant excisions, or locally advanced disease remains uncertain and requires targeted investigation. Additionally, all patients underwent planned radiotherapy, precluding assessment of scaffold performance in the absence of adjuvant treatment. Fourth, while MRI-based volumetry served as the reference standard for objective assessment, measurement variability related to imaging protocols, segmentation methods, and inter-observer differences may introduce measurement error. Although mitigated by standardized protocols and blinded dual radiologist review, this variability could impact the precision of volume retention estimates. Fifth, the absence of histopathological correlation represents a significant gap in understanding scaffold remodeling at the cellular level. While MRI integration scores provide surrogate evidence of tissue ingrowth and neovascularization, direct histologic confirmation of scaffold degradation, collagen replacement, cellular composition, and vascular density would strengthen biological interpretations and mechanistic understanding. Future studies incorporating protocol biopsies or opportunistic tissue sampling during revision surgeries would provide valuable insights.

Despite these limitations, the consistently large effect sizes across volumetric, radiologic, and patient-reported outcomes provide a strong rationale for future randomized controlled trials comparing HPTC scaffold-assisted reconstruction with conventional volume replacement strategies, with extended follow-up and broader inclusion criteria.

Clinical practice implications

The findings of this study have direct and immediate implications for contemporary BCS practice. HPTC scaffold implantation offers surgeons a regenerative volume restoration option that can be integrated into standard BCS workflows without the need for additional donor-site surgery, specialized flap expertise, or prolonged operative time. This is particularly relevant in small- to moderate-sized breasts, where even limited tissue excision can result in visible contour deformity and where traditional volume displacement techniques may be insufficient [1,6].

The ability to achieve early and stable volume restoration with preserved imaging interpretability supports the use of HPTC scaffolds in patients planned for adjuvant radiotherapy, a population in whom implants and fat grafting may be suboptimal. Most patients in this cohort underwent planned adjuvant radiotherapy according to standard oncologic protocols. Importantly, scaffold implantation did not delay adjuvant therapy. However, the long-term effects of radiotherapy on scaffold remodeling and volume stability require evaluation in extended follow-up studies. Furthermore, the favorable safety profile and absence of device-related serious adverse events suggest that scaffold implantation can be adopted without increasing perioperative risk. From a patient-centered perspective, the significant improvement in BREAST-Q scores underscores meaningful cosmetic and psychosocial benefit, aligning reconstructive success with patient expectations and quality-of-life priorities [3,14].

Future research directions

While the present study establishes early safety and efficacy, several important avenues for future research remain. Foremost among these is the need for randomized controlled trials comparing HPTC scaffold-assisted reconstruction with established techniques such as fat grafting or oncoplastic volume replacement flaps. Such trials should incorporate longer follow-up to evaluate the durability of volume retention, response to adjuvant radiotherapy, and long-term cosmetic stability.

Future studies should also explore patient selection criteria, including defect size thresholds, breast volume stratification, and integration with volume displacement techniques [19-21]. Incorporation of validated oncologic quality-of-life instruments such as the EORTC QLQ-C30 and EQ-5D would enable comprehensive assessment of functional and psychosocial outcomes [16,17].

Additionally, histologic correlation studies and advanced imaging analyses may further elucidate the mechanisms of scaffold remodeling and tissue regeneration in the breast, strengthening the biological rationale for widespread adoption.

Regulatory and economic considerations

From a regulatory standpoint, collagen-based biomaterials have an established safety record across multiple surgical disciplines, including chronic wound management and reconstructive surgery [4,5,12,13]. The absence of permanent foreign material and the predictable biodegradation profile of HPTC scaffolds may simplify regulatory pathways compared with synthetic implants or novel biologics.

Economically, scaffold-assisted volume restoration has the potential to reduce overall treatment costs by avoiding donor-site surgery, decreasing operative time, and minimizing the need for staged or revision procedures. When considered alongside reduced hospital stay, faster recovery, and improved patient satisfaction, HPTC scaffolds may represent a cost-effective adjunct to BCS. Formal health economic analyses will be essential in future trials to quantify these benefits and support broader reimbursement considerations [23,24].

## Conclusions

This prospective multicenter clinical trial demonstrates that implantation of an HPTC scaffold following breast-conserving surgery (BCS) is a safe and biologically effective strategy for immediate breast volume restoration. The intervention achieved high early volume retention, progressive radiologic integration, and substantial improvement in patient-reported cosmetic satisfaction, with no device-related serious adverse events or compromise of oncologic surveillance. The concordance between objective volumetric preservation, MRI-based evidence of tissue integration, and validated patient-reported outcomes underscores the regenerative potential of collagen-based scaffolds as an alternative to conventional volume replacement techniques. By avoiding donor-site morbidity and minimizing surgical complexity, this approach addresses key limitations associated with fat grafting, flap-based reconstruction, and implant use in partial breast defects. Although limited by its single-arm design, low sample size, and short-term follow-up, the consistently large effect sizes observed across primary and secondary endpoints provide compelling justification for future randomized controlled trials. Longer-term comparative studies are warranted to establish durability, response to adjuvant radiotherapy, and comparative effectiveness against established oncoplastic techniques. Overall, HPTC scaffold implantation represents a promising regenerative adjunct in BCS, with the potential to expand reconstructive options while preserving oncologic safety and improving patient-centered outcomes.
